# 3D X-ray Microscopy of Ultrasonically Welded Aluminum/Fiber-Reinforced Polymer Hybrid Joints

**DOI:** 10.3390/ma14071784

**Published:** 2021-04-04

**Authors:** Florian Staab, Mario Prescher, Frank Balle, Lutz Kirste

**Affiliations:** 1 Walter and Ingeborg Herrmann Chair for Power Ultrasonics and Engineering of Functional Materials (EFM), Department of Sustainable Systems Engineering (INATECH), Faculty of Engineering, University of Freiburg, 79110 Freiburg, Germany; frank.balle@inatech.uni-freiburg.de; 2 Fraunhofer Institute for Applied Solid State Physics (IAF), 79108 Freiburg, Germany; Mario.Prescher@iaf.fraunhofer.de (M.P.); Lutz.Kirste@iaf.fraunhofer.de (L.K.); 3 Freiburg Materials Research Center (FMF), 79104 Freiburg, Germany

**Keywords:** ultrasonic welding, hybrid joints, 3D X-ray microscopy, nondestructive structural analysis, fiber-reinforced polymer, segmentation by machine learning

## Abstract

Ultrasonically welded hybrid aluminum/fiber-reinforced PEEK joints were analyzed non-destructively with an X-ray microscope. The potential and limitations of the technology as a non-destructive testing method were investigated. For a quantitative evaluation, joints with suitable and unsuitable parameters were compared. For a further comparison, geometric modifications of the joining partners were made, and the influence on the structure and process variation of the resulting hybrid joints was examined on a microscopic level. By using a tool for 3D segmentation of the composition of the joining zone, quantitative information on volume-specific proportions could be obtained and compared in relation to each other.

## 1. Introduction

Current developments in lightweight design result, on the one hand, in highly specialized individual materials and, on the other hand, in a very heterogeneous mix of materials for complex structural applications. Structural components of modern civil aircraft today consist of highly engineered light metal alloys based on aluminum and titanium, suitable light strength advanced steels, tailored fiber-reinforced composites, or multi-material sandwich components [1,2]. Conventional and currently industrially used form-fit joining processes for light metal/FRP joints in multi-material design, especially rivet or screw connections, are widely used due to their high reliability and easy handling. Due to the cut-outs required for assembly, usually in the form of drilling holes in the joining partners, this joining technology significantly reduces the structural performance of the components [3,4]. The interruption or deflection of the load-bearing fiber path restricts the advantages of fiber-reinforced plastics. The resulting thickened joining surfaces and the introduction of additional masses by joining elements further reduce the lightweight potential. In addition, this process is characterized by extensive pre- and post-treatment of the joining zone, and non-destructive testing of the joining process is only possible to a limited extent [5].

The investigation of suitable low-heat joining methods for hybrid joints such as for example ultrasonic welding (USW) [6,7,8], friction stir spot welding (FSSW) [9], or adhesive bonding [10] is not the only engineering challenge. Although the joining technology of FSSW can produce a favorable mechanical interlocking between aluminum and load-bearing fibers, damage to the CFRP laminate due to fiber fracture and delaminations caused by the process was observed [11]. Adhesively bonded joints consisting of aluminum and fiber-reinforced plastics rely on additional surface pre-treatment processes and require long process times for curing [12]. The evaluation and analysis of the joint structure and the non-destructive testing methods possibly derived from it are also relevant elements towards research for modern multi-material joints. Hybrid or multi-material structures can pose challenges to analytical methods due to material differences.

Typically, techniques such as transmission (TEM) and scanning electron microscopy (SEM) are used to examine the aluminum/fiber-reinforced polymer joint structures from a microscopic perspective. Using destructive serial slices, stacks of 2D images can be collected and successively aligned to create three-dimensional (3D) volume images. However, after the analysis, the samples are destroyed, and further treatment or analysis is therefore impossible. Apart from the destructive character of this type of analysis, a fundamental challenge is to find and prepare the region of interest (ROI). For these reasons, there is a need for non-destructive structural analysis methods that are also suitable for composite materials such as aluminum/fiber-reinforced polymer joints. In this work, laboratory X-ray microscopy (XRM) was used for a non-destructive structural analysis of aluminum/fiber-reinforced polymer joints. When X-rays pass through a sample, their attenuation depends on the atomic weight of the material and the volume of the material, and the attenuation is given by the Beer–Lambert law [13]. The transmitted X-rays can be monitored, resulting in a two-dimensional projection radiograph. The principle of XRM is based on collecting a series of X-ray radiographs while rotating the sample 360°, from which a mathematical algorithm can be used to reconstruct a virtual three-dimensional depiction of the sample [14]. Subsequently, the three-dimensional data set can be used to generate nearly any virtual cross-section of the sample non-destructively. The ability to view all three planes of a cross-section allows information to be collected that would have been destroyed in a physical cross-section sample preparation for a TEM or SEM analysis. XRM analysis allows measuring density variations, which can be used to understand the structural features of as-prepared and thermally or mechanically stressed samples with a resolution of, depending on the instrument, in the µm to even tenths of a nm range. In this way, the structure of the individual composites, defects such as transversal matrix cracks, delamination of the composites, cavities, and other defects can be made visible and dimensioned. However, should TEM or SEM analysis be necessary for any reason, XRM can be incorporated into a correlative microscopy workflow to ensure finding the ROI for a highly efficient and high-quality result [15].

Not only can X-ray microscopy be used to derive a quality assurance assessment of hybrid Al/FRP joints for damage and defect analysis, but it is also a powerful tool for qualitative and quantitative evaluation of the joining zone, the influence of the joining process on the base materials, and statements on the bonding mechanism. This method is already being used in research to characterize, analyze, and evaluate for example polymer and metal matrix composite materials [16,17,18] or material composition due to advanced manufacturing or joining processes [19,20,21,22]. X-ray tomography and 3D reconstruction of µCT-measurements has been performed on bolted FRP joints to quantify failure mechanics [23] and on ultrasonically welded hybrid joints to gain structural information on a macroscopic level [8]. Previous research on the ultrasonic joining process for fiber-reinforced thermoplastics has shown that selected and controlled process parameters can have a significant influence on the overall process, at particular temperatures occurring in the joining zone and the development of the joint [24]. Non-destructive testing methods of hybrid materials and joints have so far been based on eddy current testing [25] or lock-in thermography [26].

In this work, the technology of X-ray microscopy is used to non-destructively characterize aluminum/FRP joints. In addition, changes in the joining zone caused by deviating process parameters or geometry effects were analyzed. Using segmentation by a machine learning algorithm, volume fractions could be determined on a microscopic scale.

## 2. Materials and Methods

### 2.1. Torsional Ultrasonic Welding of Light Metal/FRP Joints

Torsional ultrasonic welding has proven to be a suitable process variant of ultrasonic metal welding technology for joining dissimilar materials such as non-ferrous metals, thermoplastics, or even ceramics, but especially aluminum alloys with fiber-reinforced thermoplastics (FRP) as well. Compared to established joining methods for light metal/FRP structures, such as riveting or adhesive bonding, the ultrasonic welding technology for industrial applications has several advantages: Due to a process time of only a few seconds, the joining process can be realized much faster and does not require any time-consuming pre- and post-treatment of the joining spots. Thanks to extensive measurement technology, numerous process parameters can be recorded and evaluated online or conclusively. In contrast to the riveting process or the use of additional filler materials, there is no need to drill holes in the FRP sheets, which can significantly reduce the mechanical performance. In addition, the USW can be highly automated.

**Figure 1:**
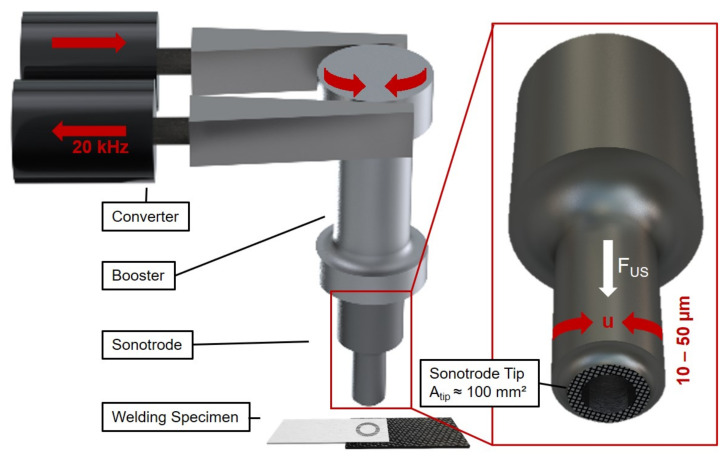
Schematic illustration of a torsional ultrasonic welding system and the welding tool (sonotrode) geometry.

A schematic illustration of a torsional ultrasonic welding system is shown in Figure 1. Basically, industrially used USW systems consist of a generator that transforms the available mains voltage of 50 Hz to the required frequency of 20 up to 100 kHz, a converter element that transfers the electrical oscillation into a mechanical oscillation by means of the piezoelectric effect, a so-called booster that stabilizes and amplifies the resulting oscillation amplitude, and the welding tool, the sonotrode, which on the one hand, introduces oscillation into the upper joining partner and, on the other hand, is used to apply the required welding force on both joining partners. The generator power integrated over time, which is necessary to maintain the oscillation at a defined displacement amplitude and applied welding force, supplies the welding energy. This is selected as an abort criterion for a joining process.

Of particular importance is the clamping device used for the realization of hybrid light metal/FRP joints with the technology of ultrasonic welding. Both joining partners are mounted independently in an ultrasonic welding-suitable jig, which allows the highest possible relative movement towards each other when introducing the oscillation during the process. Only a sufficiently high relative movement in the joining interface leads to a suitable degree of heat generation, with which the process can be performed with satisfactory results.

A special feature of this joining process for aluminum/FRP is the resulting bonding mechanism. When the oscillation is introduced into the metallic joining partner, the temperature at the interface between the two joining partners rises rapidly due to frictional effects until the melting temperature of the polymer is reached [27]. Molten polymer is displaced outwards due to the applied welding force until the reinforcing fibers, composed mainly of glass or carbon fibers, are exposed to the joining interface. Caused by the welding force and favored by the temperature increase in the joining zone, the metallic joining partner is plastically deformed and pressed into the interstices between the individual bundles and fibers. A mechanical interlocking between fibers and metal occurs in this area, which is why the overall joint fails under tensile shear load, not in its interface, but in the uppermost fiber ply [27].

Due to the kinematics of torsional ultrasonic welding, some effects relevant to the bond formation occur here. The local amplitude distribution in the welding tool tip area of a torsional oscillating sonotrode depends on the distance to the oscillation axis. With the present sonotrode geometry, the displacement amplitude at the inner edge of the ring-shaped sonotrode tip area is only 67% of the amplitude of the outer edge. This results in locally varying heat generation in the interface, which is reflected in different bonding mechanisms and local strengths. In segments of the joining zone with direct contact to the sonotrode tip, a cohesive bond primarily occurs through mechanical interlocking between the metallic joining partner and the reinforcing fibers. In areas inside and outside the characteristic sonotrode ring, melted polymer solidifies again, resulting in a significantly weaker adhesive hybrid bond [28].

### 2.2. Base Materials

**Figure 2:**
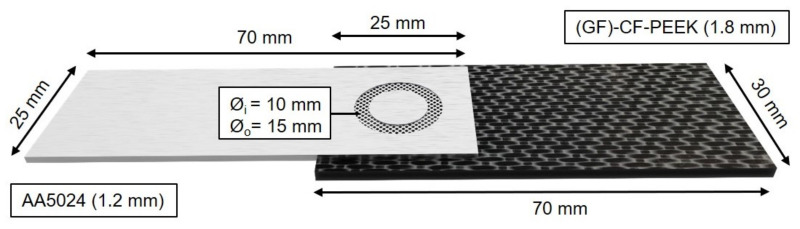
Specimen geometry of an ultrasonically welded hybrid AA5024/(GF)-CF-PEEK joint.

The subject of the 3D X-ray microscope investigations was the torsionally ultrasonically welded hybrid aluminum/FRP composites. The aluminum alloy AA5024, a AlMgSc alloy widely used for aerospace applications, was used as the metallic joining partner. Fiber-reinforced polyether ether ketone (PEEK) was used as the composite part, which consisted of six layers of carbon fibers and one ply of glass fibers for electrochemical decoupling, both in satin 1/4 fabric. The overall fiber volume ratio was V_f_ ≈ 55%, and its thickness was 1.8 mm. Both joining partners were cut from sheet material to the required sample geometry (Al: 70 × 25 mm, CFRP: 70 × 30 mm; see Figure 2), and the metallic joining partner was milled to a specified thickness of 1.2 mm. All experimental materials were provided by project partners AIRBUS (Bremen, Germany) and Composite Technology Center GmbH (Stade, Germany) and originated from aircraft manufacturing. The selected material properties are given in Table 1.

**Figure 3:**
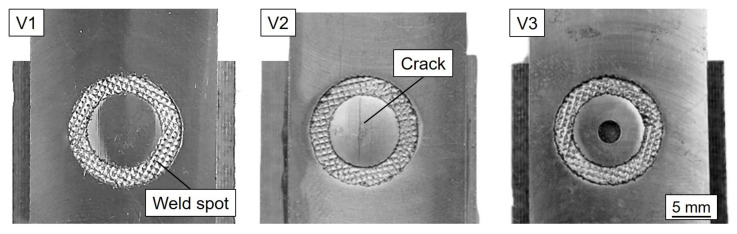
Overview for the three different AA5024/(GF)-CF-PEEK variants of Table 2.

The welding experiments were performed at 20 kHz on a torsional ultrasonic welding press Type TSP3000 from the manufacturer Telsonic Ultrasonics (Switzerland). Based on a design of experiments in a previous study [27], a welding force F_US_ = 300 N, a welding energy W_US_ = 4300 Ws, and a displacement amplitude at the outer edge u = 40 µm were selected as suitable welding parameters for the chosen material pairing. The joints failed at an ultimate tensile shear force F_UTS_ = 8310 ± 540 N. An overview of the investigated variants is given in Table 2. Specimen variants are shown in Figure 3. The measurement of F_UTS_ was based on at least six tested specimen. A self-designed anvil, which pneumatically clamped both joining partners separately, enabled the reproducible fabrication of the hybrid joint specimen of a predetermined geometry. A monolithic steel sonotrode with an annular tip surface was used as the welding tool. This had an inner diameter of 10 mm and an outer diameter of 15 mm, resulting in a tip area of about 100 mm². The sonotrode had a pyramidal tip pattern with a height of 0.4 mm, a width of 0.8 mm, and a profile angle of 45°.

In order to make a comparison of the microscopic structure between suitable and unsuitable process parameters, a joint was welded at a significantly increased welding force F_US_ = 380 N and welding energy W_US_ = 4800 Ws (Variant 2 (V2)). On the one hand, an increased temperature in the joining zone due to the increased energy input and a deviating kinematics of the bond formation due to deviating contact pressure forces were to be expected. In order to enable a varying flow behavior of the molten matrix material during the joining process, a hole with a diameter of 1.8 mm was drilled prior to the welding experiment in the center of the joining zone of the metallic joining partner for a different geometry variant (Variant 3 (V3)). It was expected that liquified thermoplastic would be able to escape from this drilling during the welding process and that, therefore, a different constitution would be produced in the joining zone.

### 2.3. X-ray Microscopy

**Figure 4:**
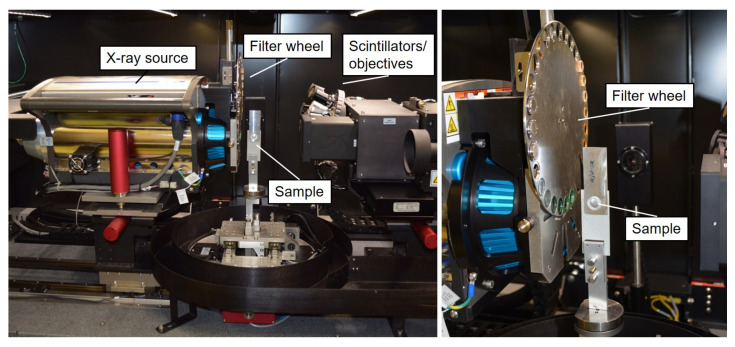
Zeiss Versa XRM-520 X-ray microscopy system at Fraunhofer IAF (used setup for Al/CFRP-joints).

X-ray microscopy (XRM) imaging was performed at the Fraunhofer Institute for Applied Solid State Physics using a Zeiss Versa XRM-520 system [30]. The XRM system displayed in Figure 4 uses a 30–160 kV polychromatic micro-focused source (tungsten target), a 2 k × 2 k CCD camera, and a tunable detector system consisting of multiple resolution and field-of-view pairings, which enables studies at a number of different spatial scales. The concept of the detector system is based on a two-stage magnification technique: The recorded images were initially enlarged by a geometric magnification. In the second step, a scintillator converted the X-rays to visible light, which was then optically magnified by an optical lens. This detection concept enables image capture with submicron resolution at even large working distances. For the Zeiss Versa XRM-520 system, the spatial resolution was ≈1 µm, and the minimum achievable voxel size was <≈ 70 nm. For the measurements, the samples were mounted in position with a metal clamp. The measurements were controlled by the Zeiss Scout-and-Scan software [30]. Different imaging regimes were used, which covered a range of voltages, depending on the required resolution of the information. The respectively used X-ray energy spectrum was tuned by the application of a filter. The data acquisition was performed by a 360° rotation. Since the samples had a flat geometry, the high aspect ratio tomography (HART) mode was used for this purpose. HART allows variable projection distances so that fewer projections were collected along the wide side of a flat sample and more along the thin side. Typically, overview scans were first performed at medium resolution, and subsequent high-resolution scans of the regions of interest were taken. The scan parameters of the different scan regimes are summarized in Table 3.

After measurement, a virtual volume for each sample was computationally reconstructed from the recorded projection images using the Zeiss XRM reconstruction software. The virtual 3D volume was essentially a 3D matrix that differentiated the sample into individual voxels. Each element of the matrix had a gray-scale value that was proportional to the atomic weight of the material within the corresponding voxel of the sample. For further data analysis, in detail the creation of slices through a virtual volume and analysis dimensions of the samples’ details, the ORS Dragonfly Pro software [31] was used. The detection and quantitative analysis in terms of the volume fractions of the individual components of the welded aluminum/FRP hybrid composites in the XRM image data were challenging due to their complexity. This required the identification of the components (aluminum, thermoplastic, CFRP, GF-textile, porosities, air inclusion, cracks) by their contrast in the images, the so-called segmentation. For the segmentation, the Adadelta algorithm was used, which was implemented in the ORS Dragonfly Pro software [31].

## 3. Results and Discussion

### 3.1. X-ray Microscopy on the AA5024/(GF)-CF-PEEK Joint Welded with Suitable Process Parameters

**Figure 5:**
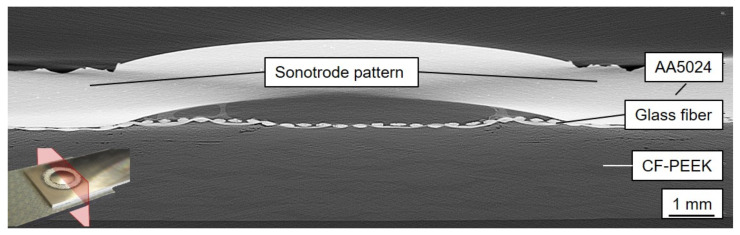
XRM slice (cross-section) along the joining area of an untreated specimen with suitable welding parameters.

Figure 5 shows an X-ray microscopy scan along an ultrasonically welded AA5024/(GF)-CF-PEEK joint. Due to plastic deformation and thermal expansion, a bulging of the metallic joining partner could be detected within both characteristic sonotrode tip contact areas. Melted thermoplastic accumulated in the edges of the bulge and solidified there again, which could be observed as a blistered structure in this region. These sections made a measurable, but comparatively small contribution to the overall strength of the joint. The brighter glass fiber top layer of the organic sheet was deformed, but not destroyed in this area and below the sonotrode tip due to the applied welding force and the resulting polymer flow. In the area below the bulged aluminum and the glass fiber textile, a few small voids in the matrix system were isolated and visible. However, since these could be localized in the area of the thermoplastic flow and were not below the outer edge of the sonotrode tip area where, as expected, the highest temperatures were present in the joining region (T_max,V1_ = 478 °C [27]), their formation was not caused by a locally exceeded decomposition temperature, but by the movement of liquified polymer.

**Figure 6:**
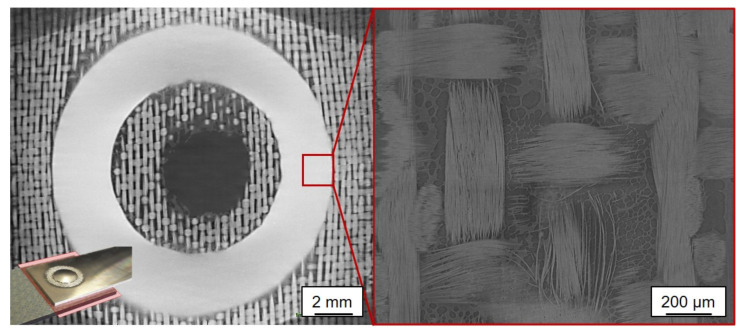
XRM cut (longitudinal) in the joining area of an untreated specimen with suitable parameters (**left**) and the detailed magnification of the joining area (**right**).

Figure 6 shows on the left an in-plane micrograph of the joining zone on the level of the glass fiber ply. The bright ring corresponds to the aluminum that was forced into this area. In the dark area in the center of the joining zone, the glass fibers lie below this plane (see Figure 6). The influence of the welding process, which also exerted an influence outside the joining area, is particularly noticeable here, in which entire bundles of the fiber textile were stretched outwards, whereby a bundle course that was distorted outwards became visible. In the detailed micrograph on the right, individual glass filaments can be seen directly in the joining zone and remaining polymer nests in the edges of the textile undulation. Although the glass fibers were visibly mechanically affected by the process, they were not destroyed by the influences of the joining process, which was why the electrical insulation properties were still present.

### 3.2. X-ray Microscopy on the AA5024/(GF)-CF-PEEK Joint Welded with Non-Suitable Process Parameters

**Figure 7:**
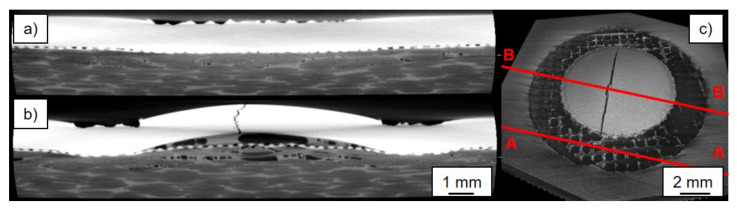
(**a**) A-A cut; (**b**) B-B cut along the joining area of a specimen welded with non-suitable parameters; (**c**) overview of the specimen.

Figure 7 shows a micrograph of an AA5024/(GF)-CF-PEEK joint welded with unsuitable parameters. Damage to the materials employed, caused by the increased energy input and excessive welding force, can be seen with the bare eye. Due to increased thermal and mechanical stress, a crack formed in the metallic joining partner, which extended over several centimeters alongside the joining zone. This increased mechanical impact due to unsuitable process parameters could also be detected geometrically: suitable parameters resulted in a bulge of the metallic joining partner of about 0.65 mm; with unsuitable parameters, it was 0.96 mm. However, also a negative influence on the fiber-reinforced joining partner can be determined with the images of the X-ray microscopy: the pore density increased significantly in the thermo-mechanical affected area of the joining zone and accumulated mainly directly in the center of the sonotrode ring (see Figure 7a,b). This can be explained, on the one hand, by the increased aluminum bulge, which in this case could absorb considerably more polymer. At the same time, the increased parameter values caused a significantly greater thermal (T_max,V2_ = 513 °C) polymer flow within the joining zone. Again, the pores were not created by exceeding the decomposition temperature. These would otherwise be located in areas with direct sonotrode contact (Figure 7a). In comparison to a joint welded with suitable parameters, the glass fiber textile here followed the curvature of the aluminum much more closely, which under certain circumstances additionally promoted the formation of polymer-poor regions and therefore pores.

### 3.3. X-ray Microscopy on Mechanically Treated AA5024/(GF)-CF-PEEK

**Figure 8:**
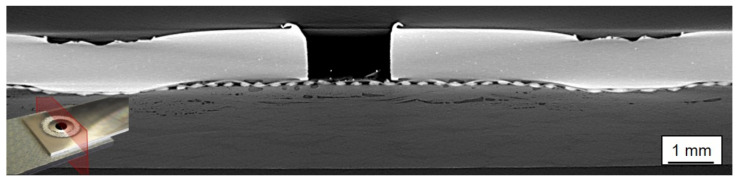
XRM slice (cross-section) along the joining area of the mechanically treated specimen (central drilled hole).

For the third variant, the influence on the polymer flow and thermal deformation of an alternative specimen geometry of the metallic joining partner was investigated. For this purpose, an AA5024/(GF)-CF-PEEK joint was welded, whose aluminum sheet was provided with a 1.8 mm drill hole before the joining process centrally in the intended joining zone. The corresponding X-ray micrograph is shown in Figure 8. The first finding was that the curvature of the metallic joining partner in the joining zone with 0.22 mm was significantly less than that of an untreated sample without a central drilled hole. This fact supported the assumption that the bulging of the metallic joining partner was primarily caused by thermal expansion through the process. The drilled hole seemed to be able to prevent buckling effectively. The glass fiber textile in the interface between the aluminum and organic sheet followed the bulging for this sample variant to a similar extent as for the joint, which was joined with unsuitable parameters. It was in continuous contact with the metallic joining partner over the entire joining zone. For this variant as well, a significantly increased pore density could be detected outside areas with direct sonotrode contact compared to the untreated specimen welded with suitable parameters. When a hole was drilled, molten polymer could propagate almost unhindered within the available space. This resulted, on the one hand, in the delocalization of the glass fiber textile and reduced the polymer content in the originally fiber-reinforced matrix, which led to pore formation.

### 3.4. Local Segmentation of the Joint Composition

**Figure 9:**
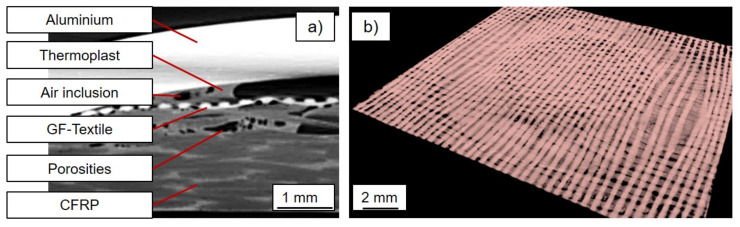
(**a**) Allocation and assignment of the individual segments of the joining zone. (**b**) Exemplary representation of one single material segment (glass fiber textile).

With the help of the Dragonfly Pro software package and its machine learning interpretation algorithm for visualizing and analyzing the measurement data, the examined section was segmented via the different contrast values of the applied materials in the joining zone. This enabled an exact determination of the volume and a localized depiction, for example of the pores. For this purpose, a distinction was made in the material segments to be detected between the carbon fiber-reinforced base material, the air inclusions therein, the glass fiber textile, the air and polymer accumulations within the forming aluminum dome, and the aluminum itself. The pores in the CFRP base material and the air entrapment in the forming aluminum dome could be identified as two different sections with the help of the algorithm. This employed assignment is displayed in Figure 9a. Figure 9b demonstrates an example of the visualization option for further analysis. In this case, only the glass fiber textile was mapped, where the characteristic displacement and shaping caused by the plastic deformation of the aluminum sheet can be seen.

The numerical values of the individual segmented volumes are given in Table 4. As indicated by the cross-sectional representations shown so far in Figure 5, Figure 7, and Figure 8, the agglomerated pore volume in the CFRP base material was lowest for the variant with suitable parameters, while the air inclusion in the forming aluminum dome was simultaneously highest. It is noteworthy that due to the increased energy input in Variant 2, significantly more polymer could be molten and was drawn into the forming aluminum dome. The corresponding volume increased by 80% from 5.40 mm³ to 9.62 mm³. Through the drilling in the center of the joining zone of the metallic joining partner, molten polymer together with glass fiber textile could penetrate into the forming dome in the third variant V3, which was why the proportion of air inclusions was lowest there. The differences in the detected volume of the glass fiber textile were remarkable as well with a 25% increase in the comparison between suitable and unsuitable parameters and about 50% in the the comparison between a mechanically untreated specimen and a drilled hole, suggesting that the glass fiber textile was decompressed in Variants 2 and 3, induced by the increased polymer mobility.

**Figure 10:**
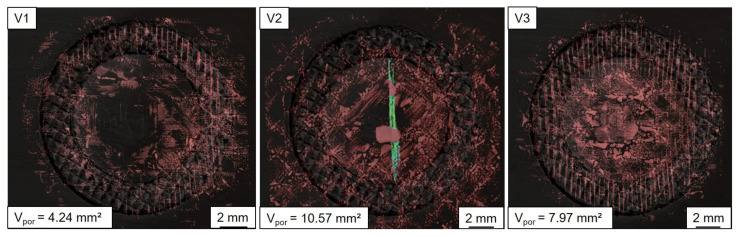
Local distribution by the joining process-induced pores (red) in the organic sheet of the three investigated specimen variants V1, V2, and V3; for V2 the position and orientation of the crack in the metallic sheet are displayed in green.

Figure 10 shows the local distribution of the pores of the three composite variants (V1: joined with suitable parameters; V2: joined with increased parameters; V3: drilled centrally before joining) together with the numerical value of the total volume. The regular linear arrangement of the air inclusions of all variants in the area of the reinforcing fibers suggested an orientation towards the individual filament bundles. Variant 1 did not allow the assumption of a local preference in the area of the sonotrode tip area. Especially in the segment inside the joining zone, pores accumulated. In the case of unsuitable welding parameters (Variant 2), in addition to the significantly larger total pore volume, a preferential accumulation in the area of the crack through the aluminum (green) could also be determined. In the case of a joint in which a hole was drilled in the metallic joining partner before the process (Variant 3), a significantly increased accumulation in the center of the sonotrode tip area could be determined.

## 4. Summary and Conclusions

Ultrasonically welded aluminum/FRP hybrid structures were analyzed and characterized with the aid of non-destructive X-ray microscopy. To test the validity of this measurement method, three different joint variants, including suitable and non-suitable parameters, as well as mechanical pre-treated joining partners, were examined, and significant qualitative differences in the constitution were determined and interpreted in a quantitative manner. The results presented in this manuscript regarding the configuration and material composition of the joining zone showed that a slight increase in the applied process parameters of about 10% of the applied welding force F_US_ and energy W_US_ already caused significant changes in the process dynamics. Although significant differences were shown in the numerical evaluation in the joining zone’s composition, the mechanical properties of the different variants did not deviate strongly. Using non-suitable parameters, the porosity in the joining area increased by more than a factor of two. In addition, volume damage caused by increased energy input and mechanical load during the joining process was detected, and modifications of the process dynamics were interpreted in detail. The 3D technology of X-ray microscopy in combination with the applied algorithms for segmentation represents a powerful quantitative and qualitative assessment tool especially for welded hybrid lightweight structures with distinguishable material specific contrasts. Using a machine learning algorithm, individual material segments of the joining zone could be examined and displayed in isolation. With this powerful method, preferred areas of pore location can be identified, as well their spatial arrangement characterized and their volume quantified on a microscopic level.

The main findings of the paper were:

- Hybrid AA5024/(GF)-CF-PEEK composites were analyzed and characterized by X-ray microscopy on a macroscopic and microscopic level. The thermomechanical influence of the joining process affected, but did not destroy the glass fiber bundles in the interface of the joining zone.
- Deviating process parameters of about 10% reduced the resulting tensile shear forces by only about 15%, but led to a qualitatively significant difference in the formation of the joining zone. The pore volume in the CFRP base material increased by 150%. The sum of the porosities and air inclusions in the forming aluminum dome remained almost identical for V1 and V2.
- By drilling a hole in the center of the joining zone, the resulting tensile shear forces were reduced by about 8%. The pore content in the CFRP base material increased by about 90%.

This non-destructive testing method could be a useful alternative to destructive testing methods such as 2D microscopy to evaluate porosities in hybrid components, especially for aerospace applications. In addition, it is a possible complement to ultrasonic testing if analysis is difficult due to the diversity of the materials used. Furthermore, high-resolution imaging allows an accurate characterization of the internal structure. By means of reference samples, this technology can be used to ensure the quality of a joining process such as ultrasonic welding.

## Figures and Tables

**Table 1:** Selected mechanical and thermal properties of AA5024 and (GF)-CF-PEEK [27].

Metal Sheet	AA5024	Composite Sheet	(GF)-CF-PEEK
Young’s modulus (GPa)	72.8 ± 0.6	Young’s modulus in fiber direction (GPa)	60.1 ± 0.4
Yield strength (MPa)	315 ± 4	Ultimate tensile strength (MPa)	850 ± 28
Ultimate tensile strength (MPa)	395 ± 5	Melting Point (°C)	340 [29]
Ultimate elongation A_50_ (%)	12.4 ± 1.2	Decomposition Temperature (°C)	>550 [29]

**Table 2:** Overview of the investigated variants of AA5024/(GF)-CF-PEEK-joints.

	Variant V1	Variant V2	Variant V3
	Reference	Increased Parameters	Geometry Variation
u (µm)	40	40	40
F_US_ (N)	300	380	300
W_US_ (Ws)	4300	4800	4300
F_UTS_ (N)	8310 ± 540	7237 ± 641	7639 ± 588

**Table 3:** X-ray microscopy (XRM) scan parameters used to collect the data sets.

Scan Type	Objective	No. of Projections	Voxel Size	Tension	Power	Binning	Projection Time
Overview	0.4×	2401	9.24 µm	40 kV	3 W	1	30 s
High-resol.	4×	3201	1.98 µm	40 kV	3 W	1	120 s

**Table 4:** By segmentation, the determined absolute and relative volume composition of the three investigated specimen variants: V1: F_UTS_ = 8310 ± 540 N; V2: F_UTS_ = 7237 ± 641 N; V3: F_UTS_ = 7639 ± 588 N.

	Variant 1		Variant 2		Variant 3	
	mm^3^	%	mm^3^	%	mm^3^	%
CFRP	521.30	57.51	586.64	57.45	598.58	58.09
Porosities in CFRP	4.24	0.47	10.51	1.03	7.97	0.77
GF textile	16.97	1.87	21.83	2.14	25.50	2.47
Air inclusion	14.13	1.56	7.89	0.77	0.69	0.07
Thermoplastic	5.40	0.60	9.62	0.94	2.07	0.20
Aluminum	344.48	38.00	384.60	37.67	395.64	38.39

## Data Availability

The data presented in this study are available on request from the authors.
